# Comparison of conventional and contemporary root canal disinfection protocols against bacteria, lipoteichoic acid (LTA), and lipopolysaccharide (LPS)

**DOI:** 10.1038/s41598-022-26855-y

**Published:** 2023-01-21

**Authors:** Theeb A. Alquria, Rayyan A. Alfirdous, Swati Gupta, Mauro P. Santamaria, Ingrid F. Santamaria, Ana P. M. Gomes, Naiara Tiradentes, Eduardo G. Silva, Frederico C. Martinho

**Affiliations:** 1 Department of Advanced Oral Sciences and Therapeutics, School of Dentistry, University of Maryland, Baltimore, MD USA; 2 Department of Restorative Dental Science, College of Dentistry, Imam Abdulrahman Bin Faisal University, Dammam, Saudi Arabia; 3 Prince Abdulrahman Advanced Dental Institute, Riyadh, Kingdom of Saudi Arabia; 4 College of Dentistry, University of Kentucky, Lexington, KY USA; 5 Department of General Dentistry, School of Dentistry, University of Maryland, Baltimore, MD USA; 6 Endodontic Division, Department of Restorative Dentistry, Institute of Science and Technology, São Paulo State University (Unesp), São José dos Campos, Brazil; 7 Department of Social and Pediatric Dentistry, Institute of Science and Technology, Institute of Science and Technology, São Paulo State University (Unesp), São José dos Campos, Brazil

**Keywords:** Endodontics, Root canal treatment

## Abstract

This study devised a dual-species biofilm model to investigate bacteria, lipoteichoic acid (LTA), and lipopolysaccharide (LPS) simultaneously, and compared the efficacy of conventional and contemporary disinfection protocols. Seventy single-rooted mandibular premolars were included. Fourteen teeth were negative control, and 56 teeth were infected with 3-week-old *E. faecalis* and *E. coli* GFP biofilm. Fourteen/56 teeth were positive control, with seven teeth processed for CLSM analysis and seven teeth sampled with paper points (PPs) and cryogenically ground for bacterial, LTA, and LPS analyses. Forty-two teeth were randomly divided into three groups: GWS (GentleWave system) + MIT (minimally invasive technique), PUI (passive ultrasonic irrigation) + CIT (conventional instrumentation technique), and XP-EF (XP-endo Finisher) + CIT (All, n = 14). Samples were collected before (s1) and after disinfection (s2) with PPs and after cryogenically ground (s3). CFUs were counted, and LTA and LPS were quantified with LTA-ELISA and LAL assay, respectively. XP-EF was as effective as PUI (p > 0.05). GWS + MIT was the most effective disinfection protocol against bacteria, LTA, and LPS (p < 0.05). In conclusion, PUI, XP-EF, and GWS were highly effective against bacteria, LTA, and LPS, with GWS being the most effective.

## Introduction

Endodontic infections are polymicrobial infections comprising gram-positive (G+) and -negative bacteria (G−)^1–3^. Lipoteichoic acids (LTAs) from G+ bacteria^4^ and lipopolysaccharides (LPSs) from G− bacteria^5^ are the two major virulent factors involved in endodontic infections.

LTA is a surface-associated adhesion amphiphile from G+ bacteria that is mostly released during bacterial multiplication and after bacteriolysis^4^. LTA plays an important role in the host immune response onset. It binds to toll-like receptor-2 (TLR-2)^6^ and induces inflammation with multiple cytokine secretions^4^. LTA has been detected in 100% of samples from primary and secondary/persistent infections^7–9^. High LTA contents are associated with severe periapical bone destruction^7^.

LPS is a molecule present in the outer membrane of G− bacteria^5^. It is the main virulence factor for G− bacteria. LPS has been recovered from primary and secondary/persistent endodontic infections (7–8, 10–13). It is associated with the severity of the endodontic infection^11–13^. High LPS contents is detect in patients with acute apical abscess^12,14^ clinical symptoms^11–13^, and large periapical bone destruction^11–13^. LPS and LTA shares many of its pathogenic properties^4^. LTA and LPS exhibit high inflammatory activity against periapical tissues, even when present in small amounts. Previous studies have shown that whole bacteria or their respective LPSs yield similar host responses^15,16^. Therefore, an optimal disinfection protocol should be effective against bacteria and their virulent factors.

Multiple disinfection protocols using different instrumentation and irrigation techniques failed to eliminate bacterial, LTA, and LPS infections^9,10,12,17^. Given the limited effectiveness of conventional disinfection protocols using different rotary and reciprocating files, supplemental treatments with passive ultrasonic irrigation (PUI)^8,18,19^ and photodynamic therapy (PDT)^20^ have been tested. However, we are still coming up short, and the search for an optimal disinfection protocol continues. Lately, contemporary disinfection protocols, with the use of anatomical files to maximize three-dimensional debridement such as XP-endo Finisher (XP-EF) (FKG Dentaire, La Chaus-de Fonds, Switzerland)^21–23^, and novel technology such as the GentleWave system (GWS) (Sonendo, Laguna Hills, CA, USA) for minimally invasive techniques^24–27^ have been explored for root canal disinfection. However, the literature is sparse. To date, there is no disinfection study comparing the efficacy of XP-EF, GWS, and PUI against bacteria, LTA, and LPS.

The current infection models available to evaluate disinfection have several limitations^17,28,29^. Currently, *no *in vitro infection model allows for simultaneously investigating bacteria, LPS, and LTA disinfection, and therefore, limited conclusions can be drawn from single evaluations. To overcome such limitations, this study devised a dual-species biofilm model to investigate bacteria, lipoteichoic acid (LTA), and lipopolysaccharide (LPS) simultaneously, and compared the efficacy of conventional and contemporary disinfection protocols.

## Results

**Figure 1:**
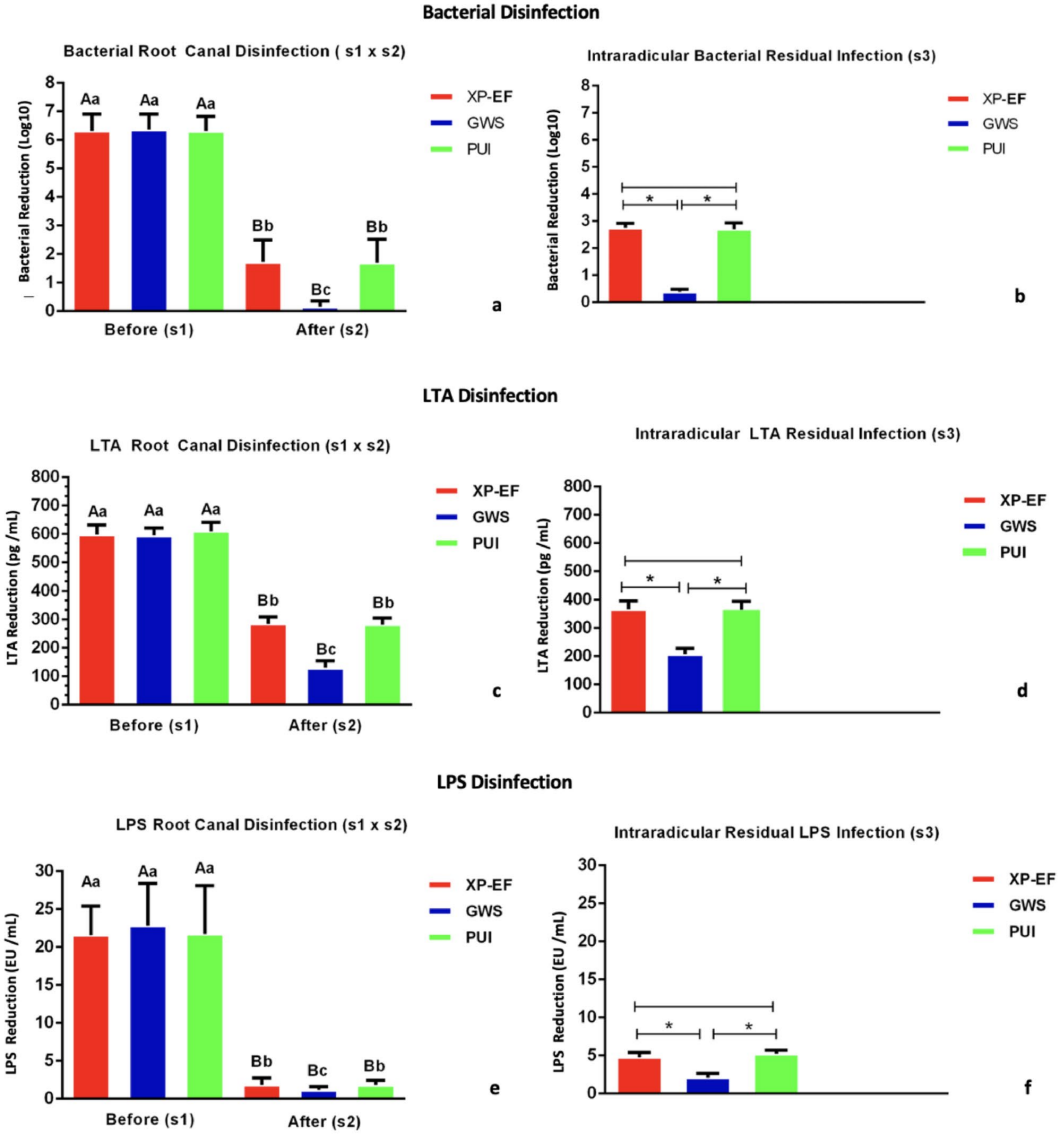
Bacterial disinfection (Log10)—(**a**) mean bacterial count recovered from the root canals before (S1) and after (s2) root canal disinfection protocols using paper point sampling. (**b**) mean intraradicular residual bacterial infection after cryogenic grinding technique (s3). Lipoteichoic acid (LTA) disinfection (pg/mL)—(**c**) mean LTA levels detected in the root canals before (S1) and after (s2) disinfection protocols using paper point sampling technique. (**d**) mean intradicular residual LTA infection after cryogenic grinding technique (s3). Lipopolysaccharide (LPS) disinfection (EU/mL)—(**e**) mean LPS levels detected in the root canals before (S1) and after (s2) disinfection protocols using paper point sampling technique; (**f**) mean intradicular residual LTA infection after cryogenic grinding technique (s3). Different uppercase letters indicate statistically significant intragroup difference. Different lowercase letters indicate statistically significant intergroup difference. Symbol (*) indicates significant difference (p < 0.05). XP-EF, XP-Endo Finisher; GWS, GentleWave System; PUI, Passive Ultrasonic Irrigation.

All 14 teeth used as a negative control showed absences of the preexisting bacteria, LTA, and LPS, validating the disinfection protocol tested here. The 14 teeth used as positive control revealed the presence of infection, in which bacteria, LTA, and LPS were detected in 100% of the teeth sampled with PPs and cryogenically ground (7/7), and all teeth submitted to CLSM (7/7) showed *E. faecalis* and *E. coli* GFP in the cervical, middle, and apical third of the canal (6/6). All the sterility swab samples collected from the external surface of the teeth showed the absence of bacteria, LTA, and LPS. At s1, bacteria, LTA, and LPS were detected in 100% of root canals in the experimental groups. Figure 1 shows the bacterial count, LTA, and LPS levels recovered at s1, s2, and s3. All disinfection protocols significantly disinfected bacteria, LTA, and LPS from the root canals at s2 (p < 0.05). XP-EF was as effective as PUI (p > 0.05). GWS + MIT was the most effective disinfection protocol against bacteria, LTA, and LPS (p < 0.05). LTA and LPS were detected in 100% of the samples at s2 and s3. At s3, GWS was the most effective protocol for bacteria, LTA, and LPS intraradicular disinfection (p < 0.05).

## Discussion

Unlike previous studies evaluating disinfection protocols against bacteria^10,21–26^, LTA^27^, or LPS^11,12,28,29^ individually, here we introduce a novel dual-species biofilm model to investigate bacteria, LTA, and LPS disinfection simultaneously. The biofilm model is comprised of *E. faecalis*, a gram-positive bacterial species that contains an LTA molecule present in the outer membrane, and *E. coli,* a gram-negative bacterial species with an LPS molecule anchored in the outer membrane. LTA and LPS, when released from the bacterial cell during bacterial multiplication and after bacteriolysis, can be sampled from the root canals and quantified using specific quantitative assays. *E. faecalis* was selected because it is frequently recovered from endodontic infections and is commonly associated with secondary/persistent infections^1,7^. Although not commonly detected in root canal infections, *E. coli* and its LPS molecule are frequently used to test LPS disinfection protocols^17,28,29^. Some of the reasons include (a) *E. coli* is the gold standard for LPS studies and one of the most potent bacterial LPS; (b) *E. coli* LPS is the standard for LAL assays used for LPS quantification; and (c) *E. coli* LPS is commercially available and accessible.

Currently, for LPS infection, there are two main in vitro LPS infection models, one with whole *E. coli* bacteria^17^ inoculation into the root canals and a second with single *E. coli* LPS^29^ contamination. However, both LPS infection models have limitations. Most previous in vitro studies^17,19^ using these infection models failed to highlight the intraradicular infection, and validated the infection solely with baseline samples using passive PP samples, which are limited to the main canal. Moreover, the evaluation of LPS disinfection were also limited to PP samples after treatment. To overcome such limitations, the biofilm model introduced here allowed us to visualize and validate the intraradicular infection under CLSM. In addition, we cryogenically ground^27,30^ and pulverized the tooth to quantify the intraradicular residual infection.

**Figure 2:**
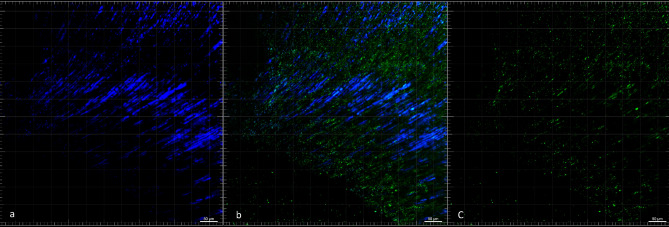
Representative CLSM image of the intraradicular infection with the dual-species biofilm model (**a**) *E. faecalis* cells in blue; (**b**) merged image; and (**c**) *E. coli* GFP (green fluorescent protein GFPmut3) cells in green. (20× Cervical third of the canal).

Different from most previous CLSM studies using LIVE/DEAD BacLight bacterial viability stain, which stains viable bacteria in red and dead in green, we designed a Live/Live experimental biofilm model, using *E. Coli* GFP visualized under CLSM in green and *E. faecalis* stained in blue. *E. coli* GFP is a clone derived from ATCC 25922 that contains a multicopy vector encoding the green fluorescent protein GFPmut3 for fluorescence imaging/detection. To the best of our knowledge, this is the first study to explore *E. coli* GFP in an endodontic infection model. After 21 days of bacterial incubation, we stained the dentin with blue fluorescent dye to highlight *E. faecalis* under CLSM (Fig. 2). To assess the spectral compatibility of the fluorescent blue dye for *E. faecalis* and *E. coli* GFP, we used a Fluorescence SpectraViewer (Thermo Fischer Scientific). The fluorescent blue dye selected to stain *E. faecalis* had an excitation at 346 nm and an emission a 442 nm, and the green fluorescent protein GFPmut3 from *E. coli* had an excitation at 505 nm and emission at 525 nm.

We compared the efficacy of conventional disinfection protocols with PUI and contemporary ones, such as the innovative XP-EF and the GWS. XP-EF is a non-tapered rotary NiTi instrument made of a unique alloy called MaxWire, which changes shape according to temperature. The file is straight in the martensitic phase at room temperature. However, when subjected to body temperature after placement inside the canal, the file enters an austenitic phase (A-phase) and assumes a unique spoon shape. This A-phase shape was designed to allow the file to access and clean areas other instruments might be unable to reach without damaging dentine or altering the canal’s original shape. This anatomical finishing file has an adaptive core technology, which presumably adapts to the root canal morphology to facilitate disinfection. Previous studies showed promising results using the XP-EF to disinfect bacteria and remove biofilm^21–23^. However, the literature is sparse on XP-EF root canal disinfection, and there is no study evaluating the effectiveness of XP-EF in disinfecting LTA and LPS.

XP-EF disinfection protocol was highly effective against bacteria, LTA, and LPS. XP-EF and PUI showed similar disinfection. From s1 to s2, XP-EF reduced mean bacteria count (Log10) from 6.31 to 1.72, LTA from 598.20 to 285.30 pg/mL, and LPS from 21.59 EU/mL to 1.83 EU/mL. The high effectiveness of XP-EF in disinfecting root canals found here agrees with previous studies^21–23^. Azim et al.^21^ found XP-EF + NaOCl was 6% more effective compared to standard needle irrigation, agitation with Endo Activator, and erbium: yttrium–aluminum–garnet laser (PIPS). Ballal et al.^22^, in a RCT, revealed that PUI and XP-EF + 2.5% NaOCl was as effective as PUI in reducing CFU counts. More recently, Cai et al.^23^ indicated XP-EF and PUI to be highly effective in disinfecting root canals with *E. faecalis* + *S. gordonii* dual-species mature biofilm by using a mixture containing 2.5% NaOCl and 9% etidronate (1-hydroxyethylidene-1,1-bisphosphonate, HEBP). PUI’s ability to improve root canal disinfection is consistent with previous studies^18,19,23–25,27^. From s1 to s2, PUI + 2.5% reduced mean bacteria count (Log10) from 6.33 to 1.69, LTA from 610.33 to 282.20 pg/mL, and LPS from 21.75 to 1.69 EU/mL. Nakamura et al.^19^ revealed bacteria reduction from 10^6^ to 10^4^ CFU/mL, LPS from 57.04 to 6.53 EU/mL. Aveiro et al.^8^ found PUI + 6% NaOCl highly effective in reducing bacteria from 10^5^ to 0 CFU/mL, LTA from 287 to 152.50 pg/mL, and LPS from 19.10 to 0.01 EU/mL. Although XP-EF and PUI were highly effective in disinfecting the root canals, both protocols failed to eliminate the infections from the root canals. More recently, Amaral et al.^31^ in a clinical study revealed the ability of XP-EF to improve root canal disinfection after instrumentation. Alfirdous et al.^32^ showed that XP-EF further improved LPS disinfection from the root canals, and the XP-EF was as effective as PUI.

**Figure 3:**
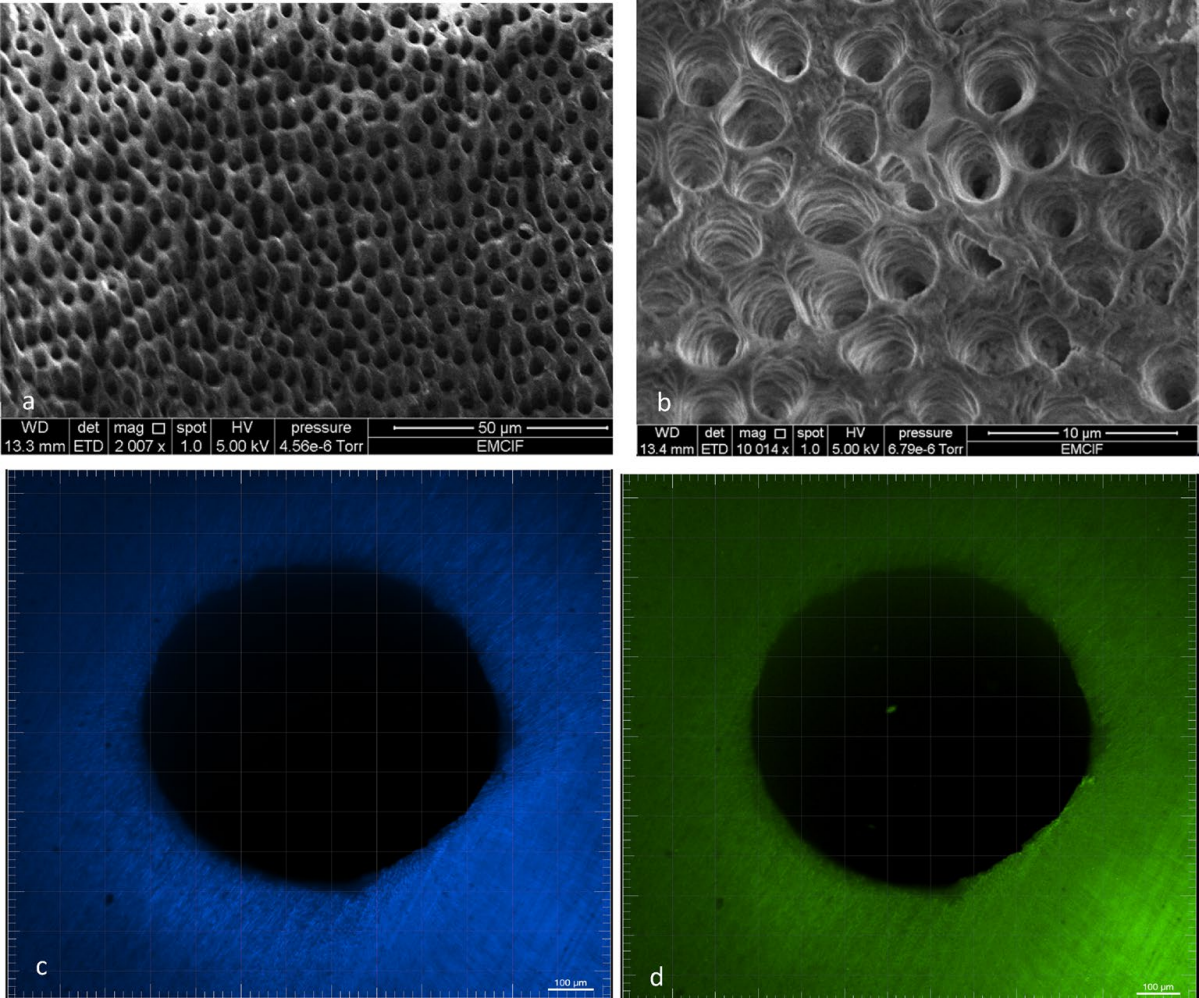
Illustrative image after GWS treatment—Scanning Electron Microscopy (SEM) image from the surface of the dentin (**a**) and the opening of the dentinal tubules (**b**) after GWS treatment; and Confocal Laser Scanning Microscopy (CLSM) (Middle third) (**c**) Blue (Excitation: 346; Emission: 442) and (**d**) Green (Excitation: 505 nm; Emission: 525 nm).

Lately, the GWS tested here has attracted clinicians’ and researchers’ interest^24–27^. GWS is a minimally invasive alternative to standard root canal treatments. Unlike ultrasonic wavelength, GWS uses multiple acoustic frequencies. The mechanism of action is described elsewhere^24,25,27^. The GWS is placed in the chamber, resting on a built occlusal platform. The implosion of microbubbles generates an acoustic field of broadband frequencies that travel through the fluid to reach the entire root canal system. GWS was the most effective protocol for root canal and intradicular disinfection. From s1 to s2, GWS reduced mean bacteria count (Log10) from 6.36 to 0.15, LTA from 595.3 to 113.8 pg/mL, and LPS from 22.84 to 1.15 EU/mL. The high effectiveness of GWS in disinfecting root canals agrees with previous studies^24–27^. Zhang et al.^24^ found GWS and PUI to be highly effective in reducing multispecies biofilm from infected root canals and bacterial DNA. The total amount of residual bacterial DNA was significantly smaller in the GWS group. A previous study demonstrated that GWS revealed a significantly greater reduction in biofilm within the mesial roots of mandibular molars and mesiobuccal roots of maxillary molars than conventional instrumental and PUI^25^. More recently, Ordinola-Zapata^26^ revealed significant shift in multispecies biofilm composition after GWS. Overall, the high effectiveness of GWS in disinfecting the root canal system found here is compatible with the cleaned surface of the dentin observed in the SEM and CLSM from a specimen after treatment (Fig. 3).

Although PP samples are routinely used for intracanal microbiological studies to evaluate root canal disinfection, they do not sample inaccessible areas like accessories, fins, dentinal tubules, or adherent biofilms. However, to overcome such limitations, we used the cryogenic grinding method to verify intradicular residual infection at s3. The cryogenic grinding method used here is similar to previous microbiological investigations^27,30,33^. Higher intraradicular residual infection was found in the XP-EF and PUI groups than in the GWS group at s3. Despite that, the findings of the cryogenically ground sample (s3) were consistent with the PP samples results (s2), with both showing the highest disinfection with GWS. The residual bacterial, LTA and LPS levels found intradicular (s3) were higher than in the main canal indicated by the PP samples (s2). Therefore, it is not unreasonable to assume that the solely PP sampling technique might underestimate the intraradicular infection levels and, consequently, the overall infection level. One limitation of the cryogenic grinding method is that it cannot be applied for the same specimen before and after treatment. However, this limitation is inherent to the cryogenic grinding method, which pulverizes the specimen.

It is important to point out that in this study teeth were randomly assigned to the experimental groups, thus tooth anatomy might have accounted for differences in the results. One of this study’s limitations was that we did not perform a quantitative CLSM analysis after treatment the study’s focus was on cryogenically grinding the roots to quantify residual intraradicular infections. Nonetheless, it is feasible to use the new infection model conceived in this study for future quantitative CLSM analyses. Another limitation of this study is that although we standardized the experimental groups with 3% NaOCl, it was not possible to normalize the irrigant volume across the MIT and CIT groups because of the different numbers of files used in each group. Moreover, the GWS tested here offers limitations inherent to the technology. The volume, flow rate, and speed at which the irrigant solutions are delivered and renewed in the root canals are not reproducible with syringe irrigation. It is not unreasonable to assume that these factors collectively might have accounted for better disinfection achieved with the GWS compared to other groups tested.

In conclusion, this study successfully devised a dual-species biofilm model to investigate bacteria, LTA, and LPS simultaneously. PUI, XP-EF, and GWS were highly effective disinfection protocols against bacteria, LTA, and LPS, with GWS being the most effective.

## Material and methods

The Institutional Review Board approved teeth collection for this study at the University of Maryland, Baltimore, MD (HP-00088564). All methods were performed following the review board at the University of Maryland guidelines and regulations. The need for obtaining informed consent was waived by the University of Maryland Baltimore ethics committee/IRB. The sample size calculation was based on a previous study^19^, with reference to a 95% confidence level, 80% power, a mean difference of 7.5, and a standard deviation of 12 in relation to the bacterial counts in Log10 CFUs^19^. G*Power (Version 3.1.9.2) was used to ensure the results attained adequate power. Random sequence generation was obtained using a computer-generated number at www.randomizer.org. Allocation concealment was conducted using the sequentially numbered, sealed, opaque envelope technique. We used 14 teeth per group in case of loss during processing. Seventy extracted human single-rooted mandibular premolars were included. All included teeth had a mature root apex, with no external or internal resorption evidence, and a single and straight canal (type I Vertucci’s classification). A radiograph was taken to ensure teeth had no prior root canal treatment, resorption, calcifications, lateral canals, or curvature. Teeth with extensive restorations, caries, crowns, cracks, or fractures were excluded. Teeth were cleaned with periodontal curettes (Hu-Friedy, Chicago, IL, USA), autoclaved for 20 min at 121 °C using a steam autoclave, and hydrated in distilled water.

All teeth were accessed with round bur # 4 and Endo Z bur. The canals were explored with a # 10 K-file (Dentsply Sirona, Charlotte, NC, USA) under a dental operating microscope (DOM) (Global Surgical Corporation, St Louis, MO, USA). Teeth with apical diameters greater than # 15 K-file were excluded. The working length (WL) was 1 mm shorter than the apical foramen. To degrade possible preexisting LPSs, all teeth were submitted to gamut radiation with cobalt 60 (20 KGy for 6 h)^17,34^.

***Dentin pretreatment—***The dentin pretreatment protocol was performed according to a previous study^27^. Briefly, all teeth were submitted to an ultrasonic bath for 10 min in 17% ethylenediaminetetraacetic acid (EDTA), followed by 5.25% sodium hypochlorite (NaOCl)^35^. The canals were soaked with 17% EDTA. The teeth were placed in a 1.5-mL plastic tube (Corning, Corning, NY) and centrifuged at 1400 g, 2000 g, 3600 g, and 5600 g in sequence for 5 min. After each cycle, the EDTA solution was renewed in the root canals. We repeated this protocol twice, and root canals were rinsed with 10 mL of 5.25% NaOCl. Teeth were placed back in the 1.5-mL plastic tube, and canals were soaked with 5.25% NaOCl and centrifuged as described above. Root canals were irrigated with 5 mL of sterile 0.5% sodium thiosulfate and rinsed off with 5 mL of sterile saline solution, and canals were dried with apryrogenic/sterile paper points (PPs).

After dentin pretreatment, 14 teeth were randomly selected as a negative control. The crown was disinfected and checked for sterility as further detailed. Canals were sampled with sterile/apyrogenic PPs, as described below, to verify the absence of preexisting root canal infections. Before cryogenic grinding, the external surfaces of the teeth were disinfected according to Siqueira^30^, and swab samples were taken from the outer surfaces of the teeth, as indicated below. All negative control samples were further processed for bacterial, LPS, and LTA analyses.

***Root canal infection—***A gram-positive bacterium, *E. faecalis* (ATCC 29212), and a gram-negative bacterium, *Escherichia coli* GFP (ATCC 25922), were used. *E. coli* GFP is a clone derived from ATCC 25922 that contains a multicopy vector encoding the green fluorescent protein GFPmut3 for fluorescence imaging/detection. A 24-h isolated pure culture of *E. faecalis* was grown on BHI agar and then suspended in 5 mL of BHI broth (BD Difico, Sparks, MD); and a 24-h isolated pure culture of *E. coli* GFP was grown on Tryptic Soy Agar (BD Difco) with 100 mcg/ml ampicillin suspended in 5 mL of BHI broth. The *E. faecalis* and *E. coli* cell suspensions were adjusted spectrophotometrically to match the turbidity of 1.5 × 10^8^ CFU/mL (equivalent to ± 0.5 McFarland standard), and all canals were inoculated. Teeth were kept in a plastic tube and incubated at 37 °C (Heratherm; Thermo Fisher Scientific, Waltham, MA). The canals were inoculated with freshly prepared BHI every two days for 21 days to obtain a 3-week-old biofilm.

Afterward, 14 infected teeth were randomly selected as a positive control for the infection. The crowns of all 14 teeth were disinfected and tested for sterility, as mentioned previously. Canals were sampled with PPs, as described below, to confirm infection. The external surfaces of the teeth were disinfected according to Siqueira et al.^30^ and tested for sterility. Seven teeth were cryogenically ground to verify the presence of bacteria, LTA, and LPS analyses as specified. The other seven teeth were processed further for confocal laser scanning microscopic analysis (CLSM).

***Confocal laser scanning microscopic analysis (CLSM)—***For the CLSM analysis, teeth were horizontally sectioned in cervical, middle, and apical thirds by using 0.3 mm Isomet discs in an Isomet 1000 Precision Cutter (Isomet Buehler, Lake Bluff, IL) cooled with sterile distilled water. The samples were placed individually on sterilized and nonpyrogenic 24-well culture plates. The Fluorescence SpectraViewer (Thermo Fischer Scientific, Waltham, MA) was used to assess the dye’s spectral compatibility to stain *E. faecalis* (Excitation: 346; Emission: 442) in blue, with *E. coli* GFP that contains a multicopy vector encoding the green fluorescent protein GFPmut3 for fluorescence (Excitation: 505 nm; Emission: 525 nm). The samples were taken to CLSM (Nikon W-1 Spinning Disk) using the 5×, 10×, and 40× oil lenses. The image samples were acquired 10 μm below the surface. Figure 2 illustrates this novel dual-species biofilm model under CLSM, with *E. faecalis* in blue and *E. coli* GFP in green.

### Root canal sampling and disinfection protocols

The root canal samples were collected before (s1), after disinfection (s2) with PPs, and after cryogenic grinding (s3), as described below.

Before root canal samples, the crown was decontaminated and disinfected with 30% H2O2 (volume/volume) for 30 s, then 5.25% NaOCl for 30 s, and then neutralized with 5% sodium thiosulfate^2,7^. The crown disinfection was checked by taking a swab sample from the crown’s surface and streaking it onto a BHI agar plate (BD Difco), incubating at 37 °C in a CO_2_ incubator for 72 h, and verified for bacterial growth. Root canal samples were conducted similar to the procedure in Louzada et al.^36^. First, for LPS investigation, the canals were sampled with sterile/apyrogenic PPs (Dentsply Sirona), which were taken to the WL, retained in position for 60 s, transferred to a sterile/apyrogenic 1.5-mL tube, and frozen at − 80 °C for further LPS analysis, as described below. Second, for bacterial investigation, three sterile/apyrogenic PPs were introduced to the WL one at a time, kept in position for 60 s, and transferred to a sterile/apyrogenic 1.5-mL tube containing 1 mL of BHI broth (BD Difco), and immediately processed for additional bacterial cultures. Lastly, one sterile/apyrogenic PP was placed to the WL, retained in position for 60 s, transferred to a sterile/apyrogenic 1.5-mL tube, and frozen at − 80 °C for further LTA analysis.

Forty-two teeth were randomly divided into three treatment groups: GWS (GentleWave system) + MIT (minimally invasive technique), PUI (passive ultrasonic irrigation) + CIT (conventional instrumentation technique), XP-EF (XP-endo Finisher) + CIT (All, n = 14). Root canals were instrumented with Vortex Blue rotary files (Dentsply Sirona) at 500 rpm. In the minimally invasive instrumentation group (MIT), the root canals were instrumented with a size # 20/0.04 Vortex Blue rotary file at the WL, while in CIT groups (conventional instrumentation technique), the canals were enlarged using a crown-down technique with a size # 35/0.06, # 30/0.06, and 25/0.06, sequentially advancing towards the WL, until a size # 35/0.04 reached the WL. The canals were irrigated with 3% sodium hypochlorite (NaOCl) after each file, and patency was checked with the # 10 hand K-file. The XP-EF, GWS, and PUI disinfection protocols tested here are described in Table 1. After root canal procedures, a second root canal sampling (s2) was conducted, as previously described.

**Table 1:** XP-EF, GWS, and PUI disinfection protocols.

Groups	Disinfection protocol
PUI	PUI was conducted according to previous study (28). Canals were rinsed with 5 mL 3% NaOCl using a 30-G side vented Maxi-i-Probe needle (Dentsply, Maillefer, Tulsa, OK). The 3% NaoCl was activated with a Satelec Sonofile K-file ultrasonic tip size # 15 (Dentsply Sirona) for 1 min at 4 power setting with a ProUltra Piezo Ultrasonic (ProUltra; Dentsply, Tulsa Dental, Tulsa, OK). The canals were irrigated with 17% EDTA for 1 min and flushed with 5 mL of 3% NaOCl. This protocol was repeated 2 times for a total of 3 min of EDTA action. After, the canals were rinsed with 5 mL 3% NaOCl and inactivated with 5 mL sterile 0.5% STS for 1 min
GWS	GWS was used as described elsewhere (28). Briefly, patency was checked, occlusal platform was built with a resin (SoundSeal, Sonendo). GWS protocol used was 3% NaOCl for 5 min, sterile distilled water (SDW) for 15 s, 17% ethylenediaminetetraacetic acid (EDTA) for 2 min, and SDW for 15 s. After GWS protocol, the root canals were irrigated with 5 mL sterile 0.5% sodium thiosulfate (STS) for 1 min and rinsed with sterile saline solution (SSL)
XP-EF	For XP-EF, a XP-EF file size # 25/0.00 (FKG, La Chaux-de-Fonds, Switzerland) was placed in a contra-angle handpiece VDW silver motor (VDW, Munich, Germany). The XP-EF was cooled down (Endo-Ice; Coltene, Cuyahoga Falls, OH) and removed in rotation mode, from the plastic tube by applying a lateral movement. Each root canal was filled with 3% NaOCl and the XP-EF was inserted, in rotation mode, into the canal. The XP-EF (800 rpm) was activated for 1 min using slow, gentle lengthwise movements. The canals were irrigated with 17% EDTA for 1 min and flushed with 5 mL of 3% NaOCl. This protocol was repeated 2 times for a total of 3 min of EDTA action. After, the canals were rinsed with 5 mL 3% NaOCl and inactivated with 5 mL sterile 0.5% STS for 1 min. * XP-EF instrumentation was conducted at 37 °C to simulate in vitro the body temperature. For that, teeth were vertically positioned and fixed in a 1.5 mL plastic tube placed in a water bath at 37 °C

PUI,  passive ultrasonic irrigation; GWS, GentleWave system; XP-EF, XP-Endo finisher.

***Cryogenic grinding technique for intraradicular disinfection analysis***—To investigate remaining bacteria, LTA, and LPS intraradicular infections, the crown was sectioned, and the root was cryogenically ground and pulverized (s3). The protocol for cryogenic grinding is reported elsewhere^27^. Briefly, teeth were individually placed in a cylindrical stainless tube inside the 6770 SPEX SamplePrep Freezer/Mill (Spex, Metuchen, NJ). We immersed the tube containing the teeth in liquid nitrogen throughout the grinding cycle. The freezer/mill machine cools samples to cryogenic temperatures and pulverizes them by magnetically shuttling a steel impactor back and forth against two stationary end plugs. The cryogenic grinding included the following protocol: (a) precooling for 10 min; (b) the first cycle, in which the samples were ground for 1 min at a rate of 10 cycles per second; (c) cooling for 1 min; (d) the second cycle, in which the samples were ground for 1 min; (e) cooling for 1 min; and (f) the third cycle, in which the samples were ground for 1 min. The total grinding time was 15 min.

All collected s1, s2, and s3 samples were processed for bacterial, LTA, and LPS analyses.

### Bacterial, LTA, and LPS analyses

***Bacterial Culture [colony-forming unit (CFU) count]—***The method for CFU/mL count has been previously reported^17^. Briefly, the bacterial samples were vortexed for 1 min and diluted with BHI broth by tenfold serial dilution to 10^–4^. Fifty microliters of each dilution were streaked in BHI agar, incubated at 37 °C for 48 h, and then the CFU was counted.

***LTA Quantification (ELISA kit)—***LTA was quantified using an LTA enzyme-linked immunoassay (LTA ELISA) kit (My BioSource, San Diego, CA) according to previous investigations^27^. LTA samples were reconstituted with buffer (phosphate-buffered saline) containing a 0.1% Tween 20 solution and 1 μL of protease inhibition cocktail (Sigma-Aldrich, Saint Louis, MO). Samples were mixed thoroughly for 60 s under vortex agitation and centrifuged at 10,000 g for 5 min. An LTA ELISA was conducted following the manufacturer’s instructions. The optical density was determined using a microplate reader set to 450 nm. Each absorbance value, expressed as mean and ± SD, was obtained, and the minimum detectable is 0.06 ng/mL (60 pg/mL).

***LPS Quantification (KQCL test)—***The chromogenic kinetic test used for the quantification of LPS was the Kinetic Chromogenic LAL Assay (KQCL test) (LONZA, Walkersville, MD), as described elsewhere^10^. The LPS quantification was conducted according to the manufacturer’s instructions. Endotoxin samples were reconstituted with 1 mL of LAL water (LAL water, LONZA). The amount of LPSs in the sample was calculated based on a polynomial-curve-fitting model generated by the WinKQCL software (BioWittaker, Cambrex C0, Walkersville, MD).

### Statistical analysis

Data were typed onto a spreadsheet and analyzed using GraphPad Prism Software (Version 6.01; GradPad Software, San Diego, CA). A Shapiro–Wilk test was used to verify the normal distribution of the data. We used a paired t-test to analyze the intragroup differences between bacterial count, LTA, and LPS levels, and we used a one-way ANOVA for intergroup analysis. For all referred tests, the significance level was set at 5%.

## Data Availability

All data generated or analysed during this study are included in this published article.
